# Vestibular recovery and central compensation after acute unilateral vestibulopathy – changes in saccadic response patterns and gains over time

**DOI:** 10.3389/fneur.2026.1832160

**Published:** 2026-04-24

**Authors:** Julia Sjögren, Per-Anders Fransson, Mikael Lars-Åke Karlberg, Måns Magnusson, Fredrik Tjernström

**Affiliations:** Department of Clinical Sciences, Otorhinolaryngology and Head and Neck Surgery, Lund University, Skåne University Hospital, Lund, Sweden

**Keywords:** AUVP, covert saccades, overt sacccades, recovery, vestibular neuritis, an indicator for vestibular loss

## Abstract

**Introduction:**

Acute unilateral vestibulopathy (AUVP), or vestibular neuritis, disrupts the vestibulo-ocular reflex (VOR), leading to impaired gaze stability during head movements. Vestibular function typically recovers to some extent. However, if recovery is incomplete, corrective eye saccades must compensate for the deficient VOR function. The aim was to analyze the progression of VOR gain recovery and development of different corrective eye saccade patterns after AUVP.

**Methods:**

A prospective longitudinal study was performed, including 43 patients with AUVP, that were examined using the video head impulse test (vHIT) during the first week after symptom onset and after 1 month, 3 months, and 12 months. Data collected included ipsilesional and contralesional gain recovery and the presence of covert and overt saccades.

**Results:**

Both ipsilesional (p < 0.001) and contralesional (p ≤ _0.038) VOR gain improved significantly over 12 months, with the most pronounced recovery occurring within the first 3 months (*p* < 0.001). The presence of covert (*p* = 0.005) and overt (*p* < 0.001) saccades decreased over time. The lower the VOR gain, the more covert saccades were present. With intermediate VOR gain there were more overt saccades, and with a gain close to normal no corrective saccades were present. Correlation analysis revealed strong relationships between lower gains on the lesion side with lower gains on the healthy side.

**Discussion:**

VOR recovery manifests with variable patterns. The degree of VOR gain deficit appears to drive the specific type of compensatory saccade patterns employed. Unilateral vestibular impairment may result in bilateral overt saccades, whereas bilateral covert saccades are rarely observed. The presence of covert saccades is a strong indicator of significant underlying VOR hypofunction.

## Introduction

The Vestibulo-Ocular Reflex (VOR) helps to maintain a stable gaze during head movements. Its effectiveness is generally measured by a parameter called gain, defined as the ratio of eye velocity to head velocity during a head turn. Ideally, the gain of the rotational VOR is 1.0, indicating that the eye movement is exactly equal and of opposite direction to the head movement (1). Acute unilateral vestibulopathy (AUVP), also known as vestibular neuritis (2), is a sudden unilateral loss of vestibular function, that leads to a deficient VOR. When the VOR fails to stabilize gaze holding, other mechanisms such as quick eye movements (corrective saccades) will have to be activated in order to redirect the target image to the fovea. These corrective saccades occur in the direction of the VOR and can be categorized as either covert (occurring while the head is still moving) or overt (occurring after the head has stopped moving) (3). Overt saccades are easily observable by an examiner without specialized equipment, whereas covert saccades are too difficult to discern by the naked eye, and thus, require a high-resolution camera with a high sample rate to be detected. In recent years, the video head impulse test (vHIT) has emerged as a highly effective objectively quantitative tool for assessing vestibular function in both the acute and chronic phases of AUVP, enabling the detection of both overt and covert saccades (4, 5). A reduced VOR gain, combined with the presence of corrective saccades, is widely recognized as a hallmark of VOR hypofunction (2). Over time after AUVP, the VOR hypofunction can either be restored (6) or continuously compensated for by corrective saccades that bring the eyes back on target (3). It has been demonstrated that covert saccades are initiated earlier during active head turns (i.e., when the patient controls the head movement) (7, 8), or when the head motion is predictable during passive head turns (i.e., when an operator moves the head) (9, 10), suggesting that the origin of such saccades might be anticipatory. The result of these early generated saccades are smaller gaze-position errors and a gaze stability close to normal in patients with unilateral vestibular loss (11, 12). It has been proposed that the hallmark of a favorable vestibular compensation is a transition from the initial saccade response, a mix of covert and overt saccades, to predominantly covert saccades (13–15).

Most studies on corrective saccades have focused on investigating the responses to a stable complete vestibular loss, that is, after vestibular schwannoma surgery. Our study aimed to track the progression of VOR gain and development of different corrective saccadic patterns following AUVP, where some degree of vestibular recovery would be expected, over the course of 1 year.

## Methods

### Patients with AUVP

Participants aged 18 to 80 were recruited from the Department of Otorhinolaryngology, Head and Neck Surgery, at Skåne University Hospital, Sweden. The diagnosis of AUVP was established based on the following criteria: a sudden onset of vertigo, without associated auditory or neurological symptoms and confirmed unilateral vestibulopathy. Diagnostic indicators included spontaneous, contralesional horizontal-torsional nystagmus that remained direction-fixed regardless of gaze and intensified without visual fixation, along with an ipsilesional pathological head impulse test (clinical Head Impulse Test), where by the naked eye detectable saccades were regarded as a sign of pathology. Importantly, there were no signs of acute central neurological dysfunction, such as central oculomotor or vestibular abnormalities, pronounced skew deviation, gaze-evoked nystagmus, or acute audiological or otological symptoms (16). These criteria align with those defined by the Bárány Society (2), except for the requirement of symptoms lasting more than 24 h. However, the classification is consistent with the term “AUVP in evolution” as described in the same framework. Patients who developed episodic symptoms during the study period were excluded. Participants were also required to have the capacity for independent decision-making and were excluded if they had a history of vertiginous disorders or if their symptoms had begun more than 48 h before potential inclusion. A comprehensive list of inclusion and exclusion criteria is provided in the appendix (Table A1).

### Ethical approval

The study protocol, registered on clinicaltrials.gov and EUdraCT, received approval from the regional scientific ethics committee and adhered to the principles outlined in the Declaration of Helsinki (Dnr 2015/5, EPN, Lund University, Sweden). All participants provided written informed consent prior to enrollment.

### Study design

Participants were randomized in blocks of six to one of three treatment arms: placebo, a 3-day corticosteroid regimen, or a 10-day corticosteroid regimen. As previously reported (17), corticosteroid treatment had no significant effect on vestibular function recovery or subjective well-being. Accordingly, no subgroup analyses based on treatment allocation were performed in the present study. Antiemetic medication was permitted during the first 2 weeks as needed, with participants advised using the lowest effective dose to support vestibular compensation. All participants received verbal and written instructions on the importance of vestibular exercises and were encouraged to initiate them as soon as possible.

The study focused on the restoration of vestibular function measured with vHIT at disorder onset (within 1 week) and at 1 month, 3 months and 12 months post-onset. Eye and head movements were recorded using the EyeSeeCam vHIT system (Interacoustics A/S, Middelfart, Denmark) at a sampling rate of 220 Hz. Before data analysis, signal processing was applied to increase the effective sampling frequency from 220 HZ to 1,000 Hz (18). The participant was seated in an armless chair positioned 1.5 meters from a white wall, where a 3 × 3 cm blue marker was placed at eye level to serve as a visual fixation point during head impulse testing. Participants were instructed to maintain visual fixation on the marker throughout the assessment. All head impulse tests were conducted by two experienced examiners, who stood behind the participant and manually applied rapid head rotations. All six semi-circular canals were examined but only data from the lateral canals will be presented in this article. The peak head velocity exceeded 150°/s, with accelerations and decelerations typically ranging from 3,000 to 8,000°/s^2^. The total head movement was approximately 10–25°. Testing continued until the software registered at least 10 accepted passive head impulses in each direction, based on the specified criteria.

### Data analysis

The gain of the VOR response for ipsilesional and contralesional head movements was described by the Interacoustics software as three ratios of horizontal eye velocity to horizontal head velocity at 40 ms, 60 ms, 80 ms, and as one mean value of the ratio during the entire acceleration phase of the head movement using linear regression analysis. VOR gain values were obtained directly from the Interacoustics software and represent the mean values of all accepted impulses by the software at each examination for 40 ms, 60 ms, 80 ms times and for the regression, respectively.

The characteristics of the saccades in the vHIT data were determined using customized software (Labview). Initially, the investigators performed a screening to exclude trials with artifacts in the eye position trace. This included removing trials affected by eye blinks, noise due to failed pupil detection or segmentation by the recording software, anticipatory eye movements, and slippage of the goggles, as indicated by eye movements occurring before the head movement onset (19). Approximately 60% of the trials were discarded during this step. After this exclusion, on average four valid trials per direction remained for each subject.

The Interacoustics vHIT system captures eye and head movements through two distinct methods: eye movements are tracked via a video recording system, while head movements are measured using an accelerometer. As an initial step, it was verified that the recorded amplitudes of head and eye movements corresponded for each trace. The analysis involved computing the positional data of both the eyes and head over a 700 ms analysis window, utilizing an integration algorithm applied to the velocity data collected by both devices. This time-window included approximately 50 ms of data preceding the onset of head movement. To determine the total displacement of the head and eyes, stable start and end positions were identified, and movement distance was calculated by subtracting the final position from the initial position. To ensure accuracy, an amplitude correction was applied to the eye position data at the sample level, using head position data as a reference. This adjustment ensured that the overall trajectory of the eyes and head exhibited identical total movement. The resulting normalized eye and head position data were then used for further analysis.

**Figure 1:**
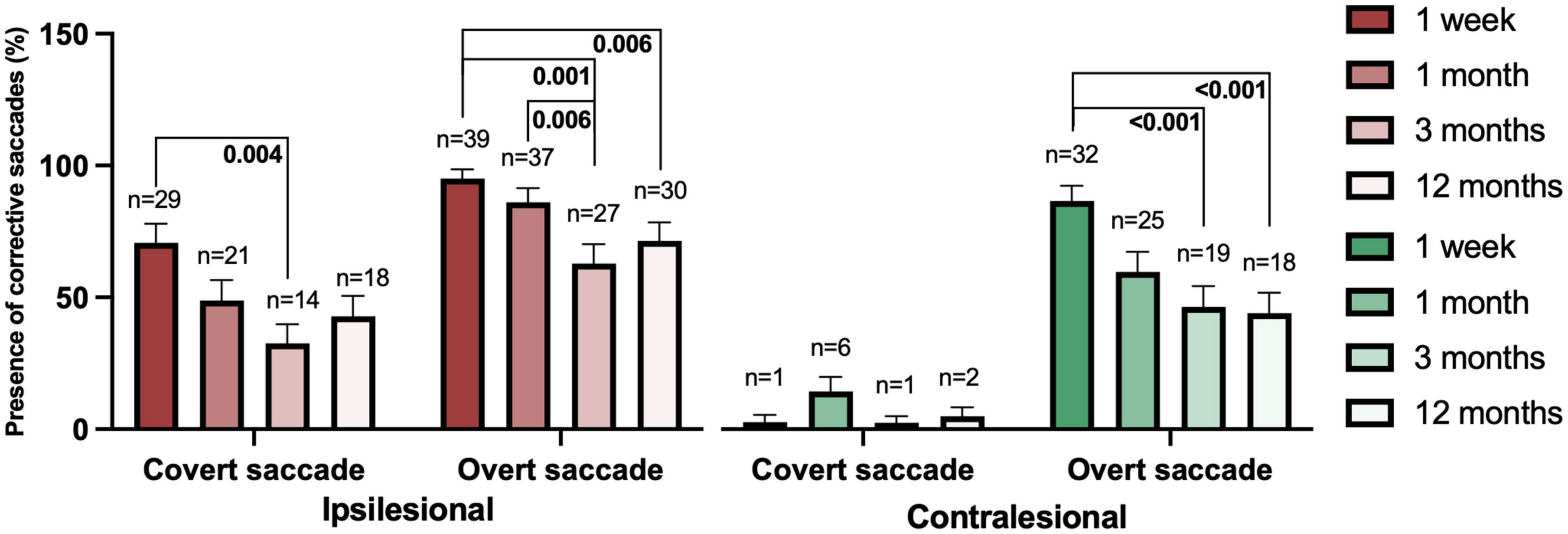
Presence of corrective saccades during ipsilesional (red) and contralesional (green) head impulses from the first week after AUVP onset to 12 months post-diagnosis in all patients (*n* = 43). Bars represent the mean value and error bars the SEM. The number of patients (*n*) exhibiting the corrective saccade at each time point is displayed above the bars. Values are presented for the individual occurrence of a saccade type, that is, a participant making a sequence of a covert and an overt saccade are included both in the covert category and the overt category.

**Figure 2:**
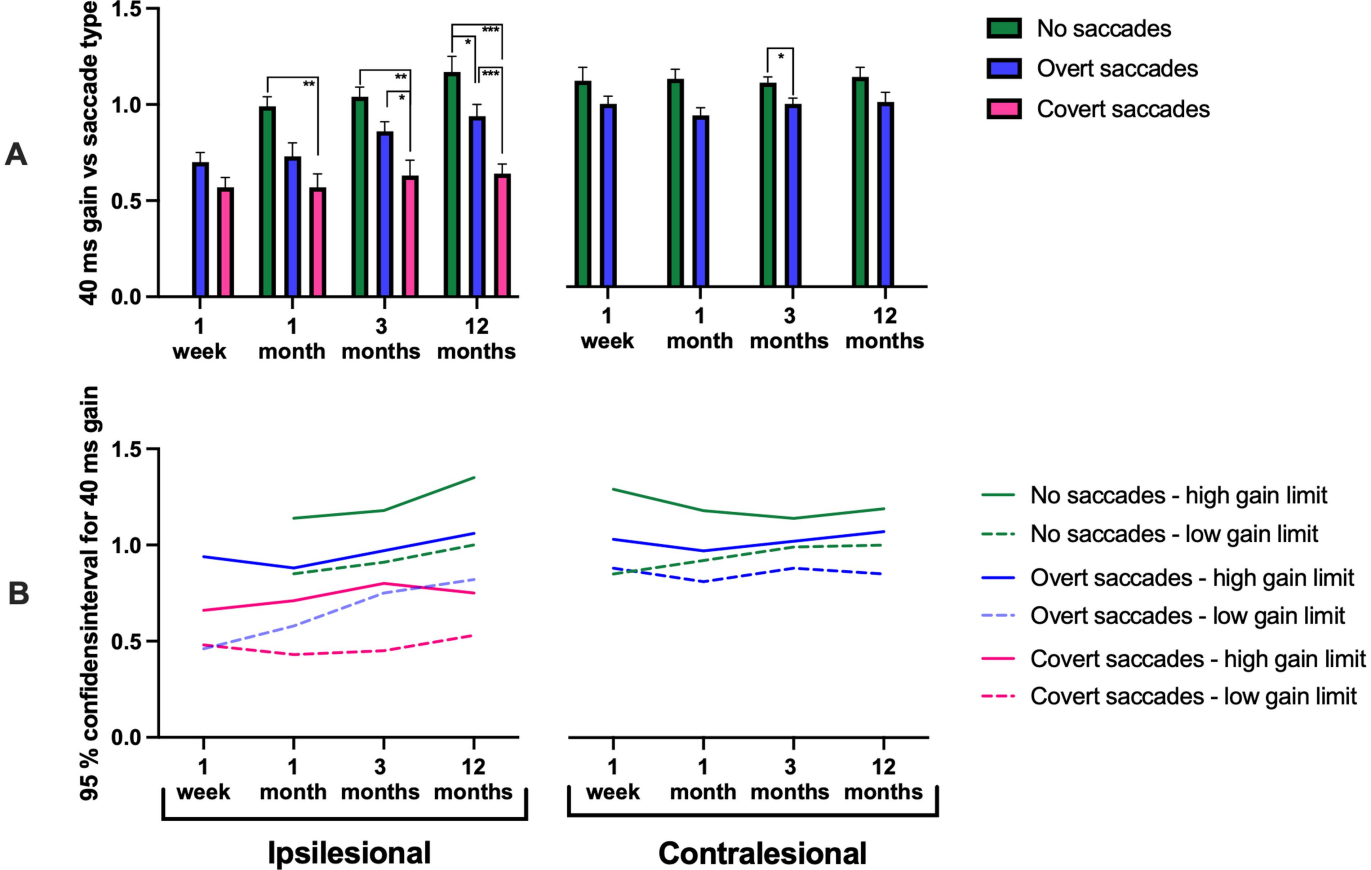
**(A)** 40 ms gain measured at disorder onset, and at 1 month, 3 months, and 12 months post-AUVP diagnosis for ipsilesional impulses (left) and contralesional impulses (right) in all patients (*n* = 43). Results are presented for three groups: patients without saccades (green), with overt saccades but no covert saccades (blue), and with covert saccades (pink). **(B)** 95% confidence intervals for 40 ms gain in patients without saccades (green), with overt saccades but no covert saccades (blue), and with covert saccades (pink). Bars represent mean values, and error bars indicate the standard error of the mean (SEM). Significant values are presented as: *p < 0.025, **p < 0.01, ***p < 0.001.

**Figure 3:**
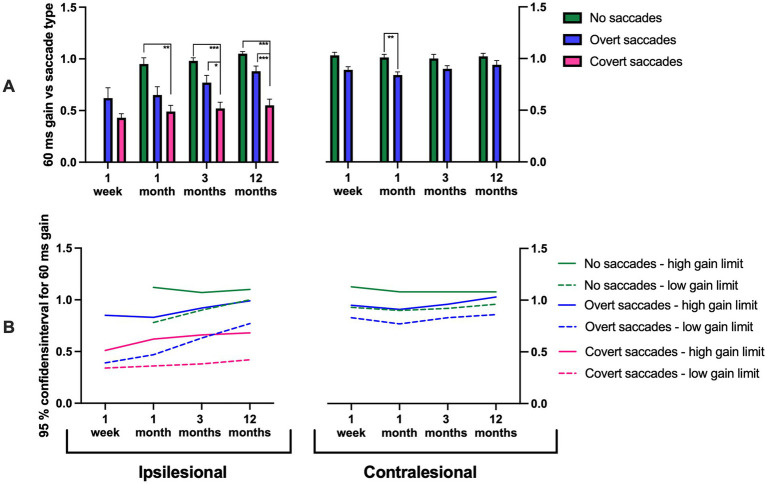
**(A)** 60 ms gain measured at disorder onset, and at 1 month, 3 months, and 12 months post-AUVP diagnosis for ipsilesional impulses (left) and contralesional impulses (right) in all patients (*n* = 43). Results are presented for three groups: patients without saccades (green), with overt saccades but no covert saccades (blue), and with covert saccades (pink). **(B)** 95% confidence intervals for 60 ms gain in patients without saccades (green), with overt saccades but no covert saccades (blue), and with covert saccades (pink). Bars represent mean values, and error bars indicate the standard error of the mean (SEM). Significant values are presented as: *p < 0.025, **p < 0.01, ***p < 0.001.

**Figure 4:**
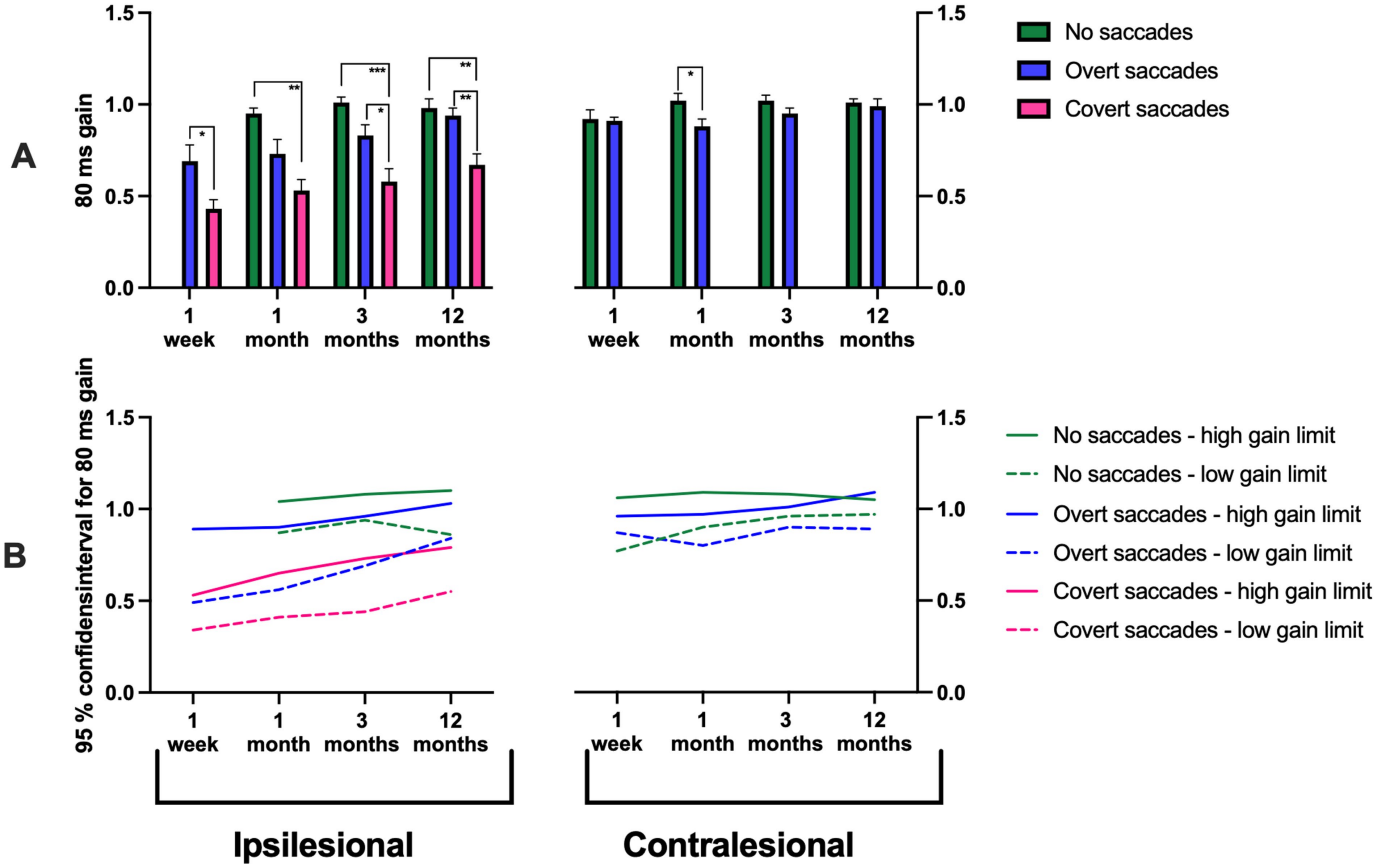
**(A)** 80 ms gain measured at disorder onset, and at 1 month, 3 months, and 12 months post-AUVP diagnosis for ipsilesional impulses (left) and contralesional impulses (right) in all patients (*n* = 43). Results are presented for three groups: patients without saccades (green), with overt saccades but no covert saccades (blue), and with covert saccades (pink). **(B)** 95% confidence intervals for 80 ms gain in patients without saccades (green), with overt saccades but no covert saccades (blue), and with covert saccades (pink). Bars represent mean values, and error bars indicate the standard error of the mean (SEM). Significant values are presented as: *p < 0.025, **p < 0.01, ***p < 0.001.

**Figure 5:**
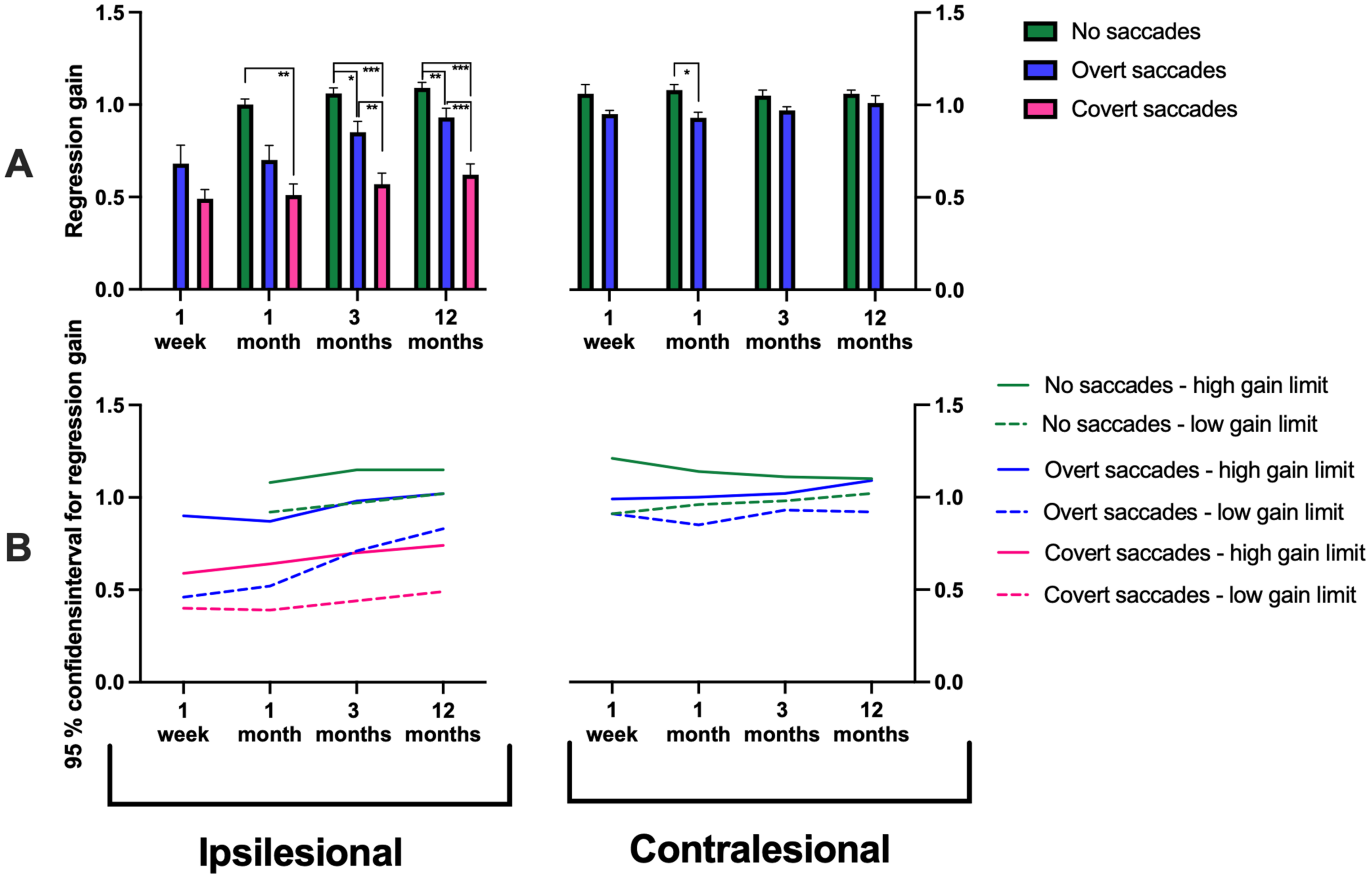
**(A)** Regression gain measured at disorder onset, and at 1 month, 3 months, and 12 months post-AUVP diagnosis for ipsilesional impulses (left) and contralesional impulses (right) in all patients (*n* = 43). Results are presented for three groups: patients without saccades (green), with overt saccades but no covert saccades (blue), and with covert saccades (pink). **(B)** 95% confidence intervals for regression gain in patients without saccades (green), with overt saccades but no covert saccades (blue), and with covert saccades (pink). Bars represent mean values, and error bars indicate the standard error of the mean (SEM). Significant values are presented as: *p < 0.025, **p < 0.01, ***p < 0.001.

Next, the software assessed whether the eye velocity data contained corrective saccades. When corrective saccades were detected, they were classified as covert saccades if initiated during the head movement, or as overt saccades if initiated after the head movement had subsided. Corrective saccades were identified based on the following criteria: (1) a peak velocity exceeding 80°/s, (2) both acceleration and deceleration phases surpassing 3,000°/s^2^, and (3) a saccade duration between 10 and 80 ms. In addition, all traces were manually inspected on raw data recording level by two experts independently to determine that the saccades detected had the correct properties and that the recordings showed no signs of artifacts like nystagmus. This manual review process was supported by providing the experts with access to both to the raw velocity data and the raw position data of the eye and head movements, and from this information could determine that any saccade made in a proper way reduced the error between eye position and head position, and thus, made the gaze more focused on the target. All covert saccades were made with the purpose of catching up with the changed head position. In contrast, overt saccades were often bidirectional, reflecting correction of both undershoot and overshoot due to imprecise eye position control during the head movement phase. At each examination time point (1 week, 1 month, 3 months, and 12 months), the presence of saccades was determined from artifact-free traces. A saccade type was classified as present if at least one artifact-free trace contained that saccade (covert or overt, respectively). Results are presented in two different ways: (1) in Figure 1, values are presented for the individual occurrence of a saccade type, that is, a participant making a sequence of a covert and an overt saccade are included both in the covert category and the overt category; (2) in Figures 2–5 values are presented for three groups: patients without making saccades (green), patients making overt saccades but no covert saccades (blue), and patients making covert saccades (pink) (which could include making also overt saccades).

### Statistics

Friedman’s test (20) was used for analyzing whether the presence of covert and overt saccades changed over repeated examinations (1 week, 1 month, 3 months, 12 months). Repeated measures GLM ANOVA (General Linear Model Analysis of Variance) was used for determining if the (dependent variables) ipsilesional and contralesional gains at 40 ms, 60 ms, 80 ms, and the regression gains were affected by the (independent within-subject variable) “Examine date” ([1 week, 1 month, 3 months, 12 months]; d.f. = 3). Repeated measures GLM ANOVA was also used for determining if the (dependent variables) ipsilesional and contralesional gains were affected by the (independent within-subject variable) “Gain time” ([40 ms, 60 ms, 80 ms]; d.f. = 2), and from the (independent between-groups variable) “Saccadic response pattern” (presence of - [covert saccades, only overt saccades, no saccades]; d.f. = 2). The GLM ANOVA analyses were performed after a validation of the appropriateness of using the statistical method, given the properties of the datasets and model residuals, e.g., that the model residuals had normal or close to normal distribution (21, 22). For the GLM ANOVA, *p* < 0.05 were considered statistically significant.

Spearman’s correlations were performed to establish whether associations existed between the four VOR gains (40 ms, 60 ms, 80 ms, regression gain) recorded on the lesion side with the four VOR gains (40 ms, 60 ms, 80 ms, regression gain) recorded on the healthy side at each of the four examinations (1 week, 1 month, 3 months, 12 months). A full factorial evaluation was performed yielding 16 correlation values per examination occasion (1 week, 1 month, 3 months, 12 months), producing a total of 64 correlation values. In the correlation analyses *p* < 0.05 were considered significant and the *R*-values were evaluated according to Cohen’s thresholds: >0.1 = small, >0.3 = moderate, >0.5 = strong and >0.7 = very strong correlation.

Non-parametric tests were employed in all *post hoc* statistical evaluations, as some datasets did not have a perfectly normal distribution. Wilcoxon matched-pairs signed-rank test (exact significance, 2-tailed) was used for within-group comparisons, specifically to analyze the evolution of gain across different examination dates and between different gain times. In the analysis of presence of saccades and changes in gain across different examination dates, *p*-values <0.017 were considered significant after Bonferroni correction. In the analysis of whether gain was different between gain time, *p* < 0.025 were considered significant after Bonferroni correction. In the Mann–Whitney U-test analysis of whether different saccadic response patterns were related to different gains, *p* < 0.025 were considered significant after Bonferroni correction.

Sample size analyses of the parameters revealed an effect size of 0.77 which shows that with the p-value set to 0.05 (2-tailed), our study requires 17 subjects to reach a power value of 0.8 for the parameter used. The statistical analyses were performed using SPSS (Version 28, IBM Corp, Armonk, New York, United States) and the power analysis was performed with GPower 3.1.9.7.

## Results

Of the 69 patients recruited, 55 were examined at the university hospital in Lund with a vHIT system found appropriate for the research task. Data from 12 patients were lost due to technical issues when transferring vHIT recordings from the Interacoustics system to our custom-made software, resulting in a final cohort of 43 participants 27 males, 16 females; mean age 55 years [standard deviation (SD) 14 years; right side lesioned – 22 participants, left side lesioned – 21 participants]. The initial vHIT assessment was performed at a mean of 3.6 days (SD 1.9) following symptom onset. Participants were evenly distributed across treatment arms, with 33% randomized to the 10-day corticosteroid regimen, 33% to the 3-day corticosteroid regimen, and 33% to placebo. Following manual data evaluation of each individual head impulse made by two co-authors independently, a total of 1,257 head impulses (658 ipsilesional and 599 contralesional) were included in the final.

**Figure 6:**
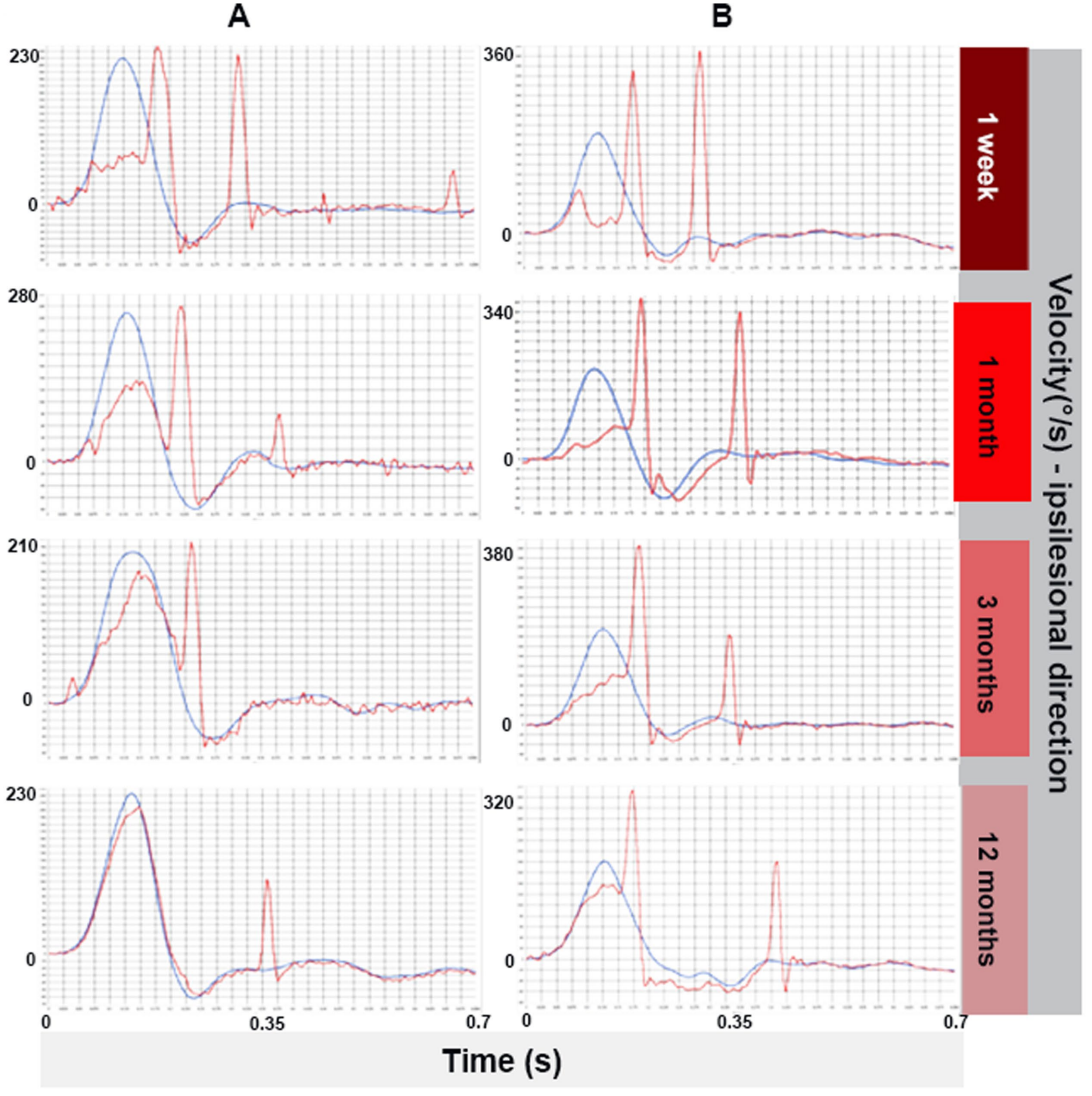
vHIT traces from two representative patients **(A,B)** with AUVP, recorded from within 1 week of disorder onset to 1-year post-lesion, during passive head impulses toward the lesioned side (ipsilesional). Head velocity is represented by the blue line, while eye velocity is shown in red. Both patients exhibit gradual gain recovery; Patient A shows an almost restored gain with a single overt saccade at 12 months, whereas Patient B demonstrates partial gain recovery with persistent covert and overt saccades throughout the 12-month period.

Figure 6 presents vHIT traces for ipsilesional head rotations in two representative patients, recorded from AUVP onset to 1-year post-onset. Initially, both patients exhibit low ipsilesional gain along with the presence of both covert and overt saccades. Over time, the ipsilesional gain gradually improves in both patients across successive examinations. After 12 months, Patient A demonstrates near-complete gain recovery with no remaining covert saccades. In contrast, Patient B shows a more limited gain improvement, with persistent covert and overt saccades observed from onset through the 12-month follow-up.

### Gain recovery

**Figure 7:**
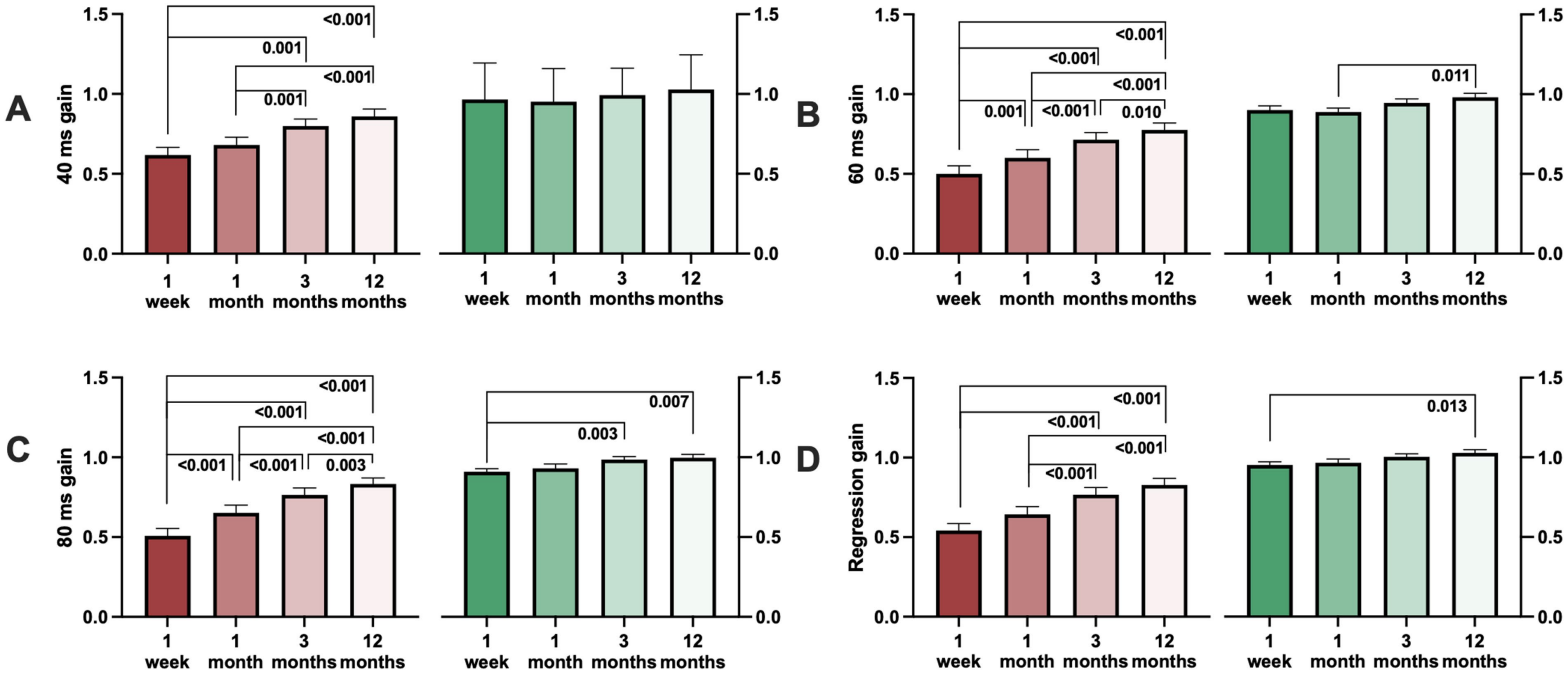
Ipsilesional gain (red) and contralesional gain (green) during the first week of onset up 1 year post AUVP diagnosis. **(A)** 40 ms gain, **(B)** 60 ms gain, **(C)** 80 ms gain, and **(D)** regression gain. Bars representing mean values and error bars indicate SEM.

Repeated-measures GLM ANOVA demonstrated a significant improvement in ipsilesional VOR gain from 1 week to 12 months across all gain time points (40 ms, 60 ms, 80 ms) and for regression gain (all *p* < 0.001) in impulses toward the lesioned side (Figure 7; Table 1). Ipsilesional gain increased from 0.62 (SD ± 0.31) to 0.86 (SD ± 0.30) at 40 ms (increased 39%); from 0.50 (SD ± 0.32) to 0.78 (SD ± 0.32) at 60 ms (increased 55%); from 0.51 (SD ± 0.29) to 0.83 (SD ± 0.24) at 80 ms (increased 63%); and from 0.54 (SD ± 0.29) to 0.83 (SD ± 0.27) for regression gain (increased 53%) (Figure 7; Table 1). In contralesional impulses, VOR gain increased significantly over repeated examinations at 60 ms (*p* = 0.038) and 80 ms (*p* = 0.013), rising from 0.90 (SD ± 0.17) to 0.98 (SD ± 0.16) at 60 ms (increasing 9%) and from 0.91 (SD ± 0.13) to 1.00 (SD ± 0.14) at 80 ms (increasing10%) (Figure 7; Table 1).

**Table 1:** Effects of examination date on gain.

v-HIT gains	Ipsilesional	Contralesional
40 ms gain	**<0.001 [29,4]**	0.473 [0,5]
60 ms gain	**<0.001 [38,8]**	**0.038 [4,9]**
80 ms gain	**<0.001 [48,5]**	**0.013 [7,4]**
Regression gain	**<0.001 [38,2]**	0.089 [3,2]

Repeated-measures GLM ANOVA with main factor “Examine date” for ipsilesional and contralesional movements. *F*-values are presented within the squared parenthesis. Bold statistics denote *p* < 0.05.

*Post hoc* with Wilcoxon analysis revealed that the most substantial gain recovery on the ipsilesional side occurred during the first 3 months (*p* < 0.001), whereas the gains on the contralesional side took longer time to recover (Figure 7).

### Presence of covert and overt saccades during AUVP recovery

During the initial examination (1 week) after onset of symptoms, covert saccades were common in ipsilesional impulses, but their presence decreased significantly from the initial to the final examination (*p* = 0.005) (Figure 1; Table 2). In contrast, contralesional impulses generated very few covert saccades, rendering statistical analysis unfeasible. Overt saccades, however, were almost equally present in ipsilesional and contralesional impulses during the first examination and their presence decreased significantly at about the same rate over time in both ipsilesional (*p* < 0.001) and contralesional impulses (*p* < 0.001) (Figure 1; Table 2).

**Table 2:** Effects of examination date on presence of corrective saccades.

Presence of saccades	Ipsilesional	Contralesional
Presence of covert saccades	**0.005**	-
Presence of overt saccades	**<0.001**	**<0.001**

Friedman’s test with main factor “Examine date.” Bold statistics denote *p* < 0.05.

Post hoc with Wilcoxon analysis revealed that the most significant reduction of both covert and overt saccades occurred within the first 3 months, followed by a stabilization of the presence of both covert and overt saccades. Similar changes were observed for ipsilesional covert saccades (*p* = 0.004), ipsilesional overt saccades (*p* = 0.001), and contralesional overt saccades (*p* < 0.001) (Figure 1).

### VOR recovery and saccadic response pattern

#### Ipsilesional gains

The repeated measures GLM ANOVA demonstrated that the ipsilesional gain values were highly related to the ipsilesional saccadic response pattern. Specifically, the ipsilesional saccadic response pattern typically included covert saccades in addition to overt saccades if the gain values were below ≈ 0.6; included overt saccades but not covert saccades if the gain values were within the range of ≈ 0.6–0.9; and included no saccades if the gain values were above ≈ 0.9 (i.e., VOR reached a complete recovery) (Figures 2–5; Table 3). This pattern was observed consistently at 1 week (*p* = 0.033), 1 month (*p* = 0.008), 3 months (*p* < 0.001), and 12 months (*p* < 0.001). At 12 months, 22% of patients exhibited no saccades, 34% exhibited only overt saccades, and 44% exhibited both covert and overt saccades.

**Table 3:** Effect of saccadic response pattern and gain time on gain.

Ipsilesional	Saccadic response pattern	Gain time	Saccadic response pattern * Gain time
1 week	**0.033 [4.9]**	**<0.001 [27.1]**	0.127 [2.4]
1 month	**0.008 [5.5]**	**0.002 [11.2]**	0.457 [0.8]
3 months	**<0.001 [12.1]**	**<0.001 [27.4]**	0.434 [0.9]
12 months	**<0.001 [20.1]**	**<0.001 [20.7]**	**0.032 [3.8]**
Contralesional			
1 week	0.264 [1.3]	0.085 [3.1]	**0.031 [5.1]**
1 month	**0.012 [7.1]**	**0.002 [11.2]**	0.520 [0.4]
3 months	**0.027 [5.3]**	**<0.001 [16.2]**	0.211 [1.6]
12 months	0.101 [2.8]	**0.020 [5.9]**	0.122 [2.5]

Repeated-measures GLM ANOVA with main factor “Saccadic response pattern” and “Gain time” and their interaction factor. *F*-values are presented within the squared parenthesis. Bold statistics denote *p* < 0.05.

**Figure 8:**
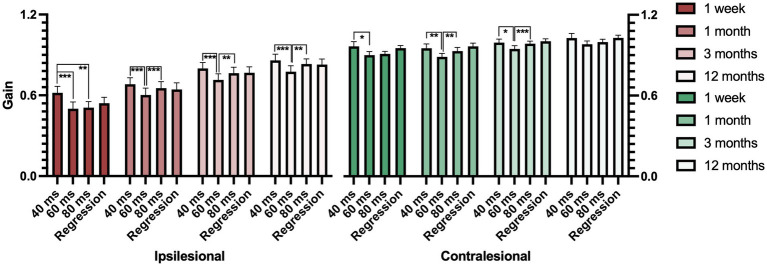
Ipsilesional gain (red) and contralesional gain (green) from the first week post-onset to 12 months after AUVP diagnosis in all patients (*n* = 43). Gain values are shown for 40 ms, 60 ms, and 80 ms time points, as well as regression-calculated gain. Bars represent mean values, with error bars indicating the standard error of the mean (SEM). Significant values are presented as: *p < 0.025, **p < 0.01, ***p < 0.001.

Additionally, there was a significant variation of the gain depending on what gain time (40 ms, 60 ms, or 80 ms) the gain was measured. The gain was typically largest at the start of the head movements at 1 week (*p* < 0.001), at 1 month (*p* = 0.002), at 3 months (*p* < 0.001), and at 12 months (*p* < 0.001) (Figure 8; Table 3).

In the ipsilesional responses at the 12-month examination, a significant interaction was observed between “saccadic response pattern” and “gain time” suggesting that the initial gain values were specifically high when the VOR responses included no saccades (*p* = 0.032).

*Post hoc* Mann–Whitney tests, evaluating the changes evoked by the saccadic response pattern, revealed that ipsilesional head movements that triggered covert saccades in addition to overt saccades had significantly lower gain, across all gain times (40 ms, 60 ms, or 80 ms) and as regression gain, at 1 month (*p* < 0.01), 3 months (*p* < 0.01), and 12 months (*p* < 0.01) compared to when the head movements triggered no saccades (Figures 2A–5A). Additionally, patients making covert saccades in addition to overt saccades had significantly lower gain, across all gain times (40 ms, 60 ns, or 80 ms) and as regression gain at 3 months (*p* < 0.05) and 12 months (*p* < 0.01) compared to patients making overt saccades (Figures 2A–5A). Additionally, patients making solitary overt saccades exhibited significantly lower regression gain compared to patients making no saccades at 3 months (*p* < 0.05), and 12 months (*p* < 0.01) follow-ups (Figures 5A).

Three distinct groups emerged and stabilized over time: patients with a high ipsilesional gain (95% CI: 1.02–1.15) exhibited no saccades, patients with moderately high ipsilesional gain (95% CI: 0.83–1.02) exhibited solitary overt saccades and finally, patients with a constant low ipsilesional gain (95% CI: 0.49–0.74) exhibited covert saccades in addition to overt saccades (Figures 2B–5B).

Post-hoc Wilcoxon analysis evaluating the changes between gain times revealed that ipsilesional gain measured at 60 ms was significantly lower than gain measured at 40 ms at 1 week (*p* < 0.001), at 1 month (*p* < 0.001), at 3 months (*p* < 0.001), and at 12 months (*p* < 0.001). Additionally, 60 ms gain was significantly lower than 80 ms gain at 1 month (*p* < 0.001), at 3 months (*p* = 0.002), and at 12 months (*p* = 0.004) (Figure 8).

#### Contralesional gains

Contralesional head rotations either elicited no saccades or produced overt saccades in both directions—opposite to and in the same direction as the head movement—reflecting correction of both undershoot and overshoot. In contrast, covert saccades were observed so infrequently that meaningful statistical analysis was not feasible.

The repeated measures GLM ANOVA demonstrated that the contralesional gain values were highly related to the contralesional saccade response pattern. Specifically, gains lower than ≈ 0.9 were associated with overt saccades, while gains higher than ≈ 0.9 were associated with an absence of saccades. This effect was observed at 1 month (*p* = 0.012) and 3 months (*p* = 0.027) (Figures 2–5; Table 3).

Furthermore, contralesional gain varied significantly depending on the gain time (40 ms, 60 ms, or 80 ms). The gain was typically largest during the start of the head movements at 1 month (*p* = 0.002), at 3 months (*p* < 0.001), and at 12 months (*p* = 0.020; Figure 8; Table 3).

A significant interaction in the contralesional responses at the 1-week examination suggested that the initial gain values were specifically high when the VOR responses included no saccades (*p* = 0.031; Table 3).

*Post hoc* Mann–Whitney tests, evaluating the changes evoked by the saccadic response pattern, revealed that for contralesional head movements, patients that made no saccades exhibited higher gains at 40 ms gain times compared to those that made overt saccades at 3 months (*p* < 0.05; Figure 2A). Moreover, patients that made no saccades exhibited higher gains at 60 ms and 80 ms gain times and as regression gain compared to those that made overt saccades at 1 month (*p* < 0.05; Figures 3A–5A). This was further reflected in the 95% CI, with patients with a gain between 1.02 and 1.10 demonstrating no saccades compared to gain values between 0.92 and 1.09 that were associated with overt saccades (Figures 2B–5B).

Post-hoc Wilcoxon analysis evaluating the changes between gain times for contralesional impulses found that the gain measured at 60 ms was significantly lower than at 40 ms at 1 week (*p* = 0.014), at 1 month (*p* = 0.009) and at 3 months (*p* = 0.014). Furthermore, 60 ms gain was significantly lower than 80 ms gain at 1 month (*p* = 0.005) and at 3 months (*p* < 0.001; Figure 8).

#### Correlation between VOR gains on lesioned side vs. VOR gains on healthy side

The correlation analyses between VOR gains on the lesion vs. healthy side revealed that at the first examination (1 week), 13 of the 16 correlations performed reached significant levels (*p* ≤ 0.037). Of these significant correlations found, six presented strong positive associations and seven moderate positive associations. Analyses of data from the second examination (1 month), revealed that 15 of the 16 correlations performed reached significant levels (*p* ≤ 0.031). Of these significant correlations found, seven presented strong positive associations and eight moderate positive associations. Analyses of data from the third examination (3 months), revealed that 16 of the 16 correlations performed reached significant levels (*p* ≤ 0.011). Of these significant correlations found, 13 presented strong positive associations and 3 moderate positive associations. Finally, analyses of data from the fourth examination (12 months), revealed that 14 of the 16 correlations performed reached significant levels (*p* ≤ 0.026). Of these significant correlations found, 2 presented strong positive associations and 12 moderate positive associations.

## Discussion

The findings of this study provide insights into the adaptive modifications of the vestibulo-ocular reflex (VOR) and saccadic eye movements following acute unilateral vestibulopathy (AUVP), that is, during a functional process of both recovery and compensation. Our results demonstrated recovery of vestibular function, that is, significant improvement of ipsilesional and contralesional VOR gains over 12 months, aligning with findings from previous studies (23, 24). The ipsilesional gain improved for all time points measured (40 ms, 60 ms, 80 ms) but only for the 60 ms and 80 ms for contralesional movements.

In our study, the number of both covert and overt ipsilesional saccades decreased over time, consistent with recent findings on AUVP (25, 26). It has been suggested that the initial saccade responses may transition from overt to covert saccades over time (14) – potentially indicating successful vestibular compensation (13, 15). However, our findings did not support such a shift of pattern for saccades. Instead, the presence of both covert and overt saccades decreased (Figure 1; Table 2). Hence, our findings suggest that covert saccades were consistently triggered when a low gain required this response – from disorder onset to 1-year post-lesion.

In our data, overt saccades were almost equally present in movements towards the contralesional side at the initial examination after onset of symptoms, which contrasts with the findings of Blödow et al. (27). This discrepancy may be attributed to differences in the identification methods for corrective saccades. In our study, corrective saccades were identified using strictly defined criteria implemented by computer software, whereas Blödow et al. (27) relied on visual inspection only. The nature of how the VOR is generated, i.e., an excitation of vestibular afferents from the side the head is rotated towards, and inhibition from the contralateral side, suggests that the saccades observed, could be evoked by a lack of inhibition and thus be purely physiological. This notion was corroborated when investigating the relationship between gains on the lesions side and the healthy side in 64 correlations - 58 significant correlations suggested that lower gains on the lesion side were associated with lower gains on the healthy side. We also found evidence for that low gains on the healthy side caused overt saccades when performing head movements towards the healthy side. This notion is also corroborated by a study examining patients with complete vestibular loss following translabyrinthine schwannoma surgery, which reported a comparable frequency of corrective saccades for contralesional impulses (28). In our cohort, overt saccades triggered in contralesional head rotations were mixed in direction, adjusting both undershoot and overshoot of the eye in respect to the focus target. Importantly, only saccades associated with minimizing the eye position error relative to the focusing target were included in the study, ensuring that only saccades making corrective movements were included and not, for example, artifacts produced by nystagmus.

Our findings demonstrate a significant relationship between VOR gain and saccadic response pattern triggered. The lowest ipsilesional gain was consistently associated with covert saccades in addition to overt saccades; intermediate gain with overt saccades and the highest gain values, corresponding with the absence of saccades. These results align with previous studies indicating that lower VOR gains are often associated with covert saccades (29). A key observation was the stability of the saccadic response pattern evoked across repeated examinations in different patient populations: (1) those with a high ipsilesional gain and no saccades (22% at 12 months); (2) those with an intermediate gain and overt saccades (34% at 12 months); (3) those with persistently low gain and covert saccades in addition to overt saccades (44% at 12 months). The 95% confidence interval values presented in Figures 2B–5B display how a certain discrete saccade response pattern vs. gain value relationship seems to have been established first when reaching the 12-month assessment. However, the figures also display two different properties of the saccade response pattern: (1) The same gain, especially during the first month, may be able to produce different saccade responses, for example, either a Covert + Overt saccade response or only an Overt saccade response; and (2) Changes in gain within large ranges may still produce the same saccade response pattern. Thus, our findings suggest that the most likely causal relationship is that, eventually, the gain level determines the kind of saccade response used. However, it may take some time before it is established what the most effective saccade response pattern is, and the optimal properties of the saccades to make.

It should be acknowledged that our participants likely consist of subgroups that had different abilities to recover from AUVP, e.g., experienced weak, moderate and excellent recovery. However, the systematic findings as presented in Figures 1–5 strongly suggest that a simpler relationship model between gain and saccade response pattern predominantly applies that is independent of subgroup behavior. As displayed in the frequency analysis presented in Figure 1, the number of patients that perform covert and overt saccades significantly decreases over time. However, despite these changes, according to the restrictive 95% confidence interval values, the participants that continue performing within certain gain ranges still respond with the same saccade response pattern. Thus, the general saccade response pattern does not change over time as an effect of a recovery by itself but is noticeably attributed to the function of the VOR, that is, the gain itself. The presence of covert saccades can directly be attributed to a low gain and persists if the gain continually remains low but ceases to exist with a better VOR.

Our results further demonstrated that the measured gain varied with the time point it was measured, with the lowest gain at 60 ms. The EyeSeeCam vHIT system supply a number of gain values with the rationale that the gain at different time points may align with certain pathology (30). Why the gain varies over the cause of the head movement acceleration remains to be further studied, in particular in patients with different disorders.

Patients with a high/normal ipsilesional and contralesional gain triggered fewer corrective saccades compared to those with an intermediate gain that triggered overt saccades and those with a low gain that triggered covert saccades in addition to overt saccades. This inverse association between covert saccade recruitment and VOR gain has previously been observed in patients with AUVP (31). In the study by Michel et al., vestibular rehabilitation initiated within 2 weeks of onset resulted in nearly restored VOR gains and the occurrence of covert saccades was significantly reduced. However, if rehabilitation began more than 2 weeks after onset, VOR gain remained low, and covert saccades occurred more frequently. The exact mechanism underlying the generation of covert saccades remains a topic of debate. For active or predictable head movements, it has been suggested that covert saccades are preprogrammed to compensate for a malfunctioning VOR, compensating the lag of the eyes relative to head movements (12). For passive head movements, where the patient cannot predict the direction of head motion, determining the underlying mechanism might be even more challenging. Retinal slip at the onset of head movement is unlikely to serve as the sole trigger for covert saccades due to their extremely rapid, almost instantaneous initiation, as well as their occurrence in complete darkness (32). Residual vestibular function also appears improbable as the sole explanation, given that covert saccades are generated even after the vestibular organ has been disconnected from the central nervous system through unilateral neurectomy (33) and covert saccades are still evoked after bilateral vestibulopathy in both light and dark conditions (34). Another possibility could be that the brain utilizes any available cues or error signals, such as sensory input from the neck at the onset of the head turn to trigger a covert saccade (35).

The restoration of VOR gain on the hypofunctional side is thought to occur through two main mechanisms. First, new connections can form as intact vestibular nerve fibers sprout and create new synapses in the deafferented vestibular nuclei, a process observed within weeks in animal models (36). Second, an increase in post-synaptic receptor density can amplify the response of remaining vestibular inputs (37, 38). The time window for these adaptive changes seems to occur within the first few months following the onset of the vestibular lesion, which have some similarities to the rehabilitation period observed after stroke (39). These changes are heavily reliant on neural input, particularly through movement, as demonstrated in vestibular rehabilitation. Notably, we observed the greatest improvements within the first 3 months, with results stabilizing thereafter. While the study did not specifically investigate the effects of vestibular rehabilitation on AUVP, and compliance with the rehabilitation protocol was not monitored, one clear finding was that the plasticity to recover gain levels and enhance performance by changing saccade response patterns seems to be mostly efficient during the first 3 months. Hence, a poorer outcome might be attributed to too low initial vestibular rehabilitation activities, poor adherence to vestibular rehabilitation and to general immobility. Another factor might be that the patient suffered from such severe damage to the vestibular organ to begin with, that these patients will be unable not fully restore their VOR, and thus, will have to continue to rely on alternative mechanisms, such as covert saccades, to maintain stable gaze.

### Limitations

This study has several limitations that should be acknowledged. While the number of patients included was reasonable, a larger sample size would have increased the statistical power and generalizability of the findings. Similarly, although additional impulses would have provided more robust data, practical constraints, such as the patients’ symptoms from AUVP and the demands of routine clinical practice, limited the number of tests performed. An unexpected challenge in this study was the high number of recordings of head impulses that contained artifacts. Despite all vHIT examinations being conducted by two highly experienced examiners and initially accepted by the Interacoustics software, many impulses were later deemed unsuitable upon closer inspection. This highlights a major limitation of the system, as reliance on automated acceptance criteria may not always ensure artifact-free data, necessitating additional manual review and potential data loss. However, only artifact-free impulses were included in the analysis, ensuring high-quality data.

Recent studies have reported that the temporal distribution of the saccades made tends to evolve from being scattered over time to becoming more controlled by appearing at identical times during and after the performed head movements (40). These findings suggest that an optimized saccadic response pattern may involve not only the broader strategy of incorporating covert or overt saccades into the VOR response, but also the fine-tuning of their timing and amplitudes. Unfortunately, the appropriate data to perform such analyses was not available for this study but it can be recommended that future research addresses this topic.

This study was focused on describing the changes that objectively can be assessed by a vHIT test, that is, the changes in gain value and whether saccades above a certain size appeared during (Covert) and after (Overt) the head movements had subsided. However, essential aspects of recovery are also whether the patient perceives vertigo and the general development of well-being during the year after an AUVP. This topic was beyond the scope of the present paper but will be addressed by future research based on the data collected during this study.

### Clinical implementation

An unambiguous VOR deficit is a key diagnostic criterion for AUVP and is most commonly identified using the clinical Head Impulse Test (HIT), in which overt saccades are taken as evidence of impaired VOR function. However, our findings suggest that overt saccades may occur across a broad range of VOR gain values (0.45–1.05), which complicates their interpretation when used in isolation. In addition, covert saccades are difficult—if not impossible—to detect with the naked eye during bedside examination. When overt saccades are observed during the manual HIT, video head impulse testing (vHIT) can provide a more detailed and quantitative assessment of their characteristics and underlying VOR function. Notably, the presence of covert saccades in addition to overt saccades indicates substantial VOR hypofunction, a finding that may be particularly useful in the diagnostic evaluation of patients with AUVP. True VOR recovery appears to be best reflected by high ipsilesional gain and the absence of corrective saccades. These findings have clinical implications, suggesting that vestibular rehabilitation should prioritize gain restoration rather than saccadic adaptation. Future studies should explore whether targeted rehabilitation can enhance VOR gain recovery while reducing reliance on compensatory saccades and to explore the potential utility of assessing covert saccades in conjunction with other clinical measures to improve diagnostic accuracy and optimize rehabilitation strategies for patients with AUVP.

## Conclusion

After AUVP, vestibular function improved over time, accompanied by a reduction in the occurrence of both covert and overt saccades. VOR recovery manifests with variable patterns. The degree of VOR gain deficit appears to drive the specific type of compensatory saccade patterns employed. Unilateral vestibular impairment may result in bilateral overt saccades, whereas bilateral covert saccades are rarely observed, as their presence is a strong indicator of severe underlying VOR hypofunction. The saccade response pattern’s ability to adjust to address different levels of vestibular functional loss, and to change if the vestibular function recovers is a likely sign of a purposefully designed adaptation.

## Data Availability

The raw data supporting the conclusions of this article will be made available by the authors, without undue reservation.
