# Supplementary material for: Vestibular recovery and central compensation after acute unilateral vestibulopathy – changes in saccadic response patterns and gains over time
Source: Front Neurol. 2026 Apr 24;17:1832160. doi: 10.3389/fneur.2026.1832160 (PMC13152781; doi:10.3389/fneur.2026.1832160)
Supplement: Supplementary file 1 [file Table_1.docx]

| **Table A1. Inclusion/Exclusion Criteria** | |
| --- | --- |
| Inclusion criteria | Age between 18 and 80 |
|  | Acute vestibular syndrome* |
|  | No dangerous HINTS* |
|  | Disease onset <48 hours |
| Exclusion criteria | New symptoms from tinnitus or hearing loss |
|  | Pregnancy |
|  | Previous gastric ulcer |
|  | Significant psychiatric disorder** |
|  | Blood pressure >180 systolic and/or >110 diastolic |
|  | Glaucoma |
|  | Serious infection or neutropenia |
|  | Chronic otitis media*** |
|  | Not fluent in Swedish**** |
|  | Incapable of making an informed decision |
| * Head Impulse test, Nystagmus, Test of Skew  ** Not excluding SSRI treatment of mild depression  *** Making caloric irrigation impossible (air irrigation not the same stimulus), **** Not possible to fill out questionnaires nor reading the written information. | |
